# Supplementary material for: HIVprotI: an integrated web based platform for prediction and design of HIV proteins inhibitors
Source: J Cheminform. 2018 Mar 9;10:12. doi: 10.1186/s13321-018-0266-y (PMC5845081; doi:10.1186/s13321-018-0266-y)
Supplement: Supplementary file 2 — Additional file 2. Source code of HIVProtI web server. [file 13321_2018_266_MOESM2_ESM.zip › HIVprotI_Source-code/hivprotI/weka-3-6-6/doc/weka/experiment/RemoteExperimentListener.html]

RemoteExperimentListener


---


|  |  |  |
| --- | --- | --- |
| **Overview** \| **Package** \| **Class** \| **Tree** \| **Deprecated** \| **Index** \| **Help** \| **Weka's home** | |  |
| **PREV CLASS**   **NEXT CLASS** | **FRAMES**    **NO FRAMES**     **All Classes** |  |
| SUMMARY: NESTED \| FIELD \| CONSTR \| METHOD | DETAIL: FIELD \| CONSTR \| METHOD |  |


---


## weka.experiment Interface RemoteExperimentListener

---

``` public interface RemoteExperimentListener ```

Interface for classes that want to listen for updates on RemoteExperiment
progress

**Version:**
:   $Revision: 1.5 $

**Author:**
:   Mark Hall (mhall@cs.waikato.ac.nz)

---

| **Method Summary** | |
| --- | --- |
| `void` | `remoteExperimentStatus(RemoteExperimentEvent e)`             Called when progress has been made in a remote experiment |

**Method Detail**

### remoteExperimentStatus

```
void remoteExperimentStatus(RemoteExperimentEvent e)
```

:   Called when progress has been made in a remote experiment

    :   **Parameters:**: `e` - the event encapsulating what happened


---


|  |  |  |
| --- | --- | --- |
| **Overview** \| **Package** \| **Class** \| **Tree** \| **Deprecated** \| **Index** \| **Help** \| **Weka's home** | |  |
| **PREV CLASS**   **NEXT CLASS** | **FRAMES**    **NO FRAMES**     **All Classes** |  |
| SUMMARY: NESTED \| FIELD \| CONSTR \| METHOD | DETAIL: FIELD \| CONSTR \| METHOD |  |


---
